# Nitrogen and chlorine co-doped carbon dots as probe for sensing and imaging in biological samples

**DOI:** 10.1098/rsos.181557

**Published:** 2019-01-23

**Authors:** Jin Li, Kai Tang, Jianxin Yu, Hanqin Wang, Mingli Tu, Xiaobo Wang

**Affiliations:** 1 Department of Reproductive Medicine, Suizhou Hospital, Hubei University of Medicine, 60 Longmen Street, Suizhou 441300, People's Republic of China; 2 Center for Translational Medicine, Suizhou Hospital, Hubei University of Medicine, 8 East Culture Park Road, Suizhou 441300, People's Republic of China

**Keywords:** carbon dots, Fe^3+^ ion, ascorbic acid, probe, biological samples

## Abstract

A facile one-step hydrothermal synthesis approach was proposed to prepare nitrogen and chlorine co-doped carbon dots (CDs) using l-ornithine hydrochloride as the sole precursor. The configuration and component of CDs were characterized by transmission electron microscopy and X-ray photoelectron and Fourier transform infrared spectroscopies. The obtained CDs (Orn-CDs) with a mean diameter of 2.1 nm were well monodispersed in aqueous solutions. The as-prepared CDs exhibited a bright blue fluorescence with a high yield of 60%, good photostability and low cytotoxicity. The emission of Orn-CDs could be selectively and effectively suppressed by Fe^3+^. Thus, a quantitative assay of Fe^3+^ was realized by this nanoprobe with a detection limit of 95.6 nmol l^−1^ in the range of 0.3–50 µmol l^−1^. Furthermore, ascorbic acid could recover the fluorescence of Orn-CDs suppressed by Fe^3+^, owing to the transformation of Fe^3+^ to Fe^2+^ by ascorbic acid. The limit of detection for ascorbic acid was 137 nmol l^−1^ in the range of 0.5–10 µmol l^−1^. In addition, the established method was successfully applied for Fe^3+^ and ascorbic acid sensing in human serum and urine specimens and for imaging of Fe^3+^ in living cells. Orn-CD-based sensing platform showed its potential to be used for biomedicine-related study because it is cost-effective, easily scalable and can be used without additional functionalization and sample pre-treatment.

## 1. Introduction

In recent years, carbon dots (CDs) have drawn tremendous attention because of their high photostability, low toxicity and good biocompatibility. These intrinsic properties give CDs numerous advantages over other optical probes (quantum dots, organic, fluorescent compounds, etc.) particularly as biosensors in biological systems [1,2]. CDs have been widely used in living cell imaging [3], fluorescent biosensing and intracellular drug delivery [4]. Until now, a number of strategies have been proposed to synthesize CDs, such as laser ablation [5], electrochemical [6], acidic oxidation [7], alkaline hydrolysis [8], pyrolysis [9], hydrothermal [10–12] and microwave [13,14]. Most of the aforementioned methods need expensive apparatus, complicated manipulation, a large amount of strong acid or alkali, which limit their realistic application. Hydrothermal method is one of the most often used techniques because it is a single-step process, easy to manipulate, cost-effective and has controlled reaction conditions. Another significant factor is the selection of appropriate precursor to prepare fluorescent CDs. It is well known that nitrogen-containing organic compounds are often used in the fabrication of CDs to improve their fluorescent properties. The rich content of nitrogen in amino acids makes them the ideal precursors for the preparation of CDs. Various amino acids have been used as materials to fabricate CDs through different methods and exhibited diverse applications in biomedical fields [15,16].

Fe^3+^ is an important essential element in various organisms. It plays vital roles in numerous biochemical activities, for instance oxygen carrier, transport and metabolism, respiratory chain, and as a cofactor in many enzyme-based catalytic reactions. Abnormal level of Fe^3+^ (deficiency or overload) will lead to anaemia, diabetes, cancer, Alzheimer's disease and heart failure [17]. Therefore, the detection and quantification of Fe^3+^ is of great importance in biological systems [18]. Traditional methods for Fe^3+^ assay rely on skilled technicians, complex measuring instruments and intricate procedures, which limit their wide applications [19–21]. To date, many reports described probes for selective detection of Fe^3+^ by fluorescence methods due to their high sensitivity, user-friendliness and on-site testing, such as organic dyes [22,23], semiconductor quantum dots [24], and metal nanoclusters [25]. Among them, most organic molecules and quantum dots are cytotoxic and often photobleaching [26]. Metal nanoclusters always need complicated synthesis procedures and are costly, which limit their prospective applications. It is still a challenge to exploit novel and biocompatible probes with low cost and sensitivity for Fe^3+^ sensing in real biological specimens (human serum, urine, living cells, etc.).

**Scheme 1.**
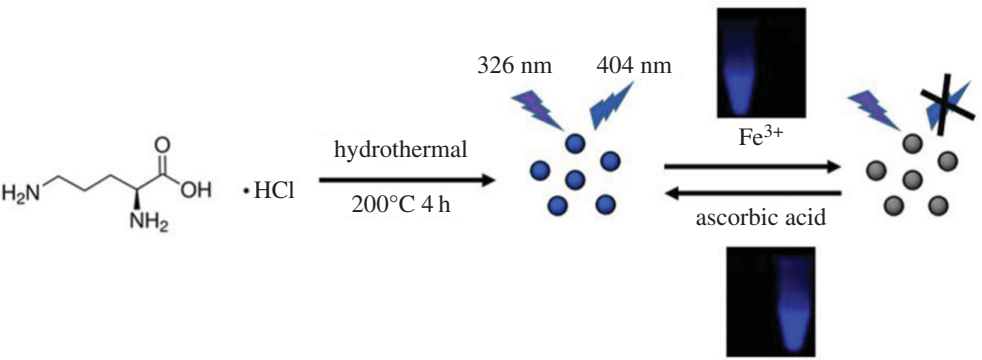
Schematic diagram of the synthesis of Orn-CDs and the detection mechanism for Fe^3+^ and ascorbic acid.

Herein, we proposed a facile one-step hydrothermal synthesis method to prepare CDs using l-ornithine hydrochloride as the only precursor (scheme 1). The as-prepared CDs (Orn-CDs) exhibit blue fluorescence with merits of low cytotoxicity, excellent photostability and water solubility. Based on a selective fluorescence off–on mechanism, a novel nanoprobe platform was developed for sensitive detection of Fe^3+^ (off) and ascorbic acid (on) with detection limits of 95.6 nmol l^−1^ and 137 nmol l^−1^, respectively. Furthermore, this chemosensor was used for the determination of Fe^3+^ and ascorbic acid in real serum and urine samples.

## 2. Experimental

### 2.1. Reagents and materials

l-Ornithine hydrochloride was obtained from Sigma-Aldrich Co. Ltd (Shanghai, China). FeCl_3_, NaCl, KCl, CaCl_2_, CuCl_2_, MgCl_2_, FeCl_2_, Zn(NO_3_)_2_, BaCl_2_, Pb(NO_3_)_2_, AlCl_3_, NiSO_4_, CoCl_2_, Cr_2_(SO_3_)_3_, CdSO_4_, Hg(NO_3_)_2_, MnCl_2_, MTT, Dulbecco's modified Eagle medium (DMEM) and quinine sulfate were purchased from Sinopharm Chemical Reagent Co. Ltd. RPMI-1640 medium was obtained from Gibco Company Ltd (USA). Fetal bovine serum was purchased from Hyclone Company Ltd (USA). A549 cells were purchased from the American Type Culture Collection (ATCC). Sephadex G-25 was purchased from GE Healthcare. All other chemical reagents were of analytical grade and used without any further purification. Ultrapure water (18.2 MΩ cm) was prepared using a Milli-Q system.

### 2.2. Instrumentation and characterization

The morphology and microstructure of CDs were characterized using FEI Technai G2 F20 transmission electron microscopy (TEM). X-ray photoelectron spectroscopy (XPS) was performed using a Thermo (EscaLab 250Xi) X-ray photoelectron spectrometer. Fourier transform infrared (FTIR) spectra were recorded by a Nicolet 5700 FTIR spectrometer with solid KBr pellets. UV–visible absorption spectra were recorded using a Shimadzu UV-2700 UV–visible spectrophotometer. The fluorescence spectra were obtained using a Horiba Fluoro Max-4 fluorescence spectrometer. Fluorescence decay spectra were measured with an FLS 980 (Edinburgh Instruments). The fluorescence images of cells were captured by an Olympus IX-73 fluorescence microscope. The cell viability was detected with a microplate spectrophotometer (Bio Tek, Epoch).

### 2.3. Preparation of Orn-CDs

l-Ornithine hydrochloride (0.25 g) was dissolved in 5 ml ultrapure water, then transferred to a Teflon-lined autoclave (50 ml) and heated at 200°C for 4 h. After hydrothermal process, the autoclave was naturally cooled to room temperature. The colourless solution turned into light brown. The obtained suspension was filtered with a 0.22 µm filter, followed by separation on Sephadex G-25 columns with ultrapure water as eluent. The fluorescent solution (approx. 100 ml) was collected, lyophilized and stored at 4°C. The obtained brown powder samples were dissolved in pure water for further study, which were denoted as Orn-CDs.

### 2.4. Determination of Fe^3+^ and ascorbic acid using Orn-CDs

The fluorescence sensing of Fe^3+^ was performed in Tris–HCl solution (20 mmol l^−1^, pH 5.5) at room temperature. Typically, 1.5 µl Orn-CDs aqueous solution (100 mg ml^−1^) was diluted to 1 ml with Tris–HCl solution (20 mmol l^−1^, pH 5.5). Then, the solution was titrated by different concentrations of Fe^3+^ (0–100 µmol l^−1^). The resulting mixture was then mixed fully and incubated for 20 min at room temperature before measurements. The fluorescence emission spectra were recorded in the wavelength range of 340 to 620 nm under excitation at 326 nm. Similarly, other metal ions were added into Orn-CDs solution to investigate the selectivity of Orn-CDs towards Fe^3+^. All experiments were performed in triplicate.

Ascorbic acid was detected in Tris–HCl solution (20 mmol l^−1^, pH 5.5) as follows. A mixed solution containing Orn-CDs (0.15 mg ml^−1^) and Fe^3+^ (200 µmol l^−1^) was prepared first. Subsequently, ascorbic acid was added to the mixture of Orn-CDs and Fe^3+^ with a final concentration from 0 to 10 µmol l^−1^, shaken thoroughly and incubated for 25 min. The fluorescence emission spectra were measured in the wavelength range of 340–620 nm under excitation at 326 nm. All measurements were run in triplicate.

### 2.5. Analysis of biological samples

The performance of the Orn-CDs for analysis of Fe^3+^ in a real sample was verified using human serum and urine obtained from the Affiliated Suizhou Hospital, Hubei University of Medicine. All specimens were diluted 100-fold by Tris–HCl solution (20 mmol l^−1^, pH 5.5) before experiment.

Fe^3+^ detection was carried out according to the following procedure. First, Orn-CDs with a final concentration of 0.15 mg ml^−1^ were added to the diluted specimen solution. The mixture was then titrated with different volumes of Fe^3+^ (1 mmol l^−1^) and mixed thoroughly. After 20 min, the emission spectra were acquired under excitation at 326 nm.

To evaluate the feasibility of Orn-CDs/Fe^3+^ sensing system for analysis of ascorbic acid in real samples, diluted samples containing CDs (0.15 mg ml^−1^)/Fe^3+^ (200 µmol l^−1^) were then titrated with different volumes of ascorbic acid (1 mmol l^−1^). After 25 min, the fluorescence spectra were acquired at 404 nm under excitation at 326 nm.

### 2.6. Live cell imaging

A549 cells were inoculated in 24-well plates with a density of 1 × 10^5^/well after incubation for 24 h at 37°C under 5% CO_2_. The cells were rinsed three times by phosphate-buffered saline. Then, 500 µl 0.5 mg ml^−1^ Orn-CDs in DMEM was added and incubated for further 4 h at 37°C. The Orn-CD-treated cells were rinsed again by phosphate-buffered saline for three times. Finally, the images of cells were captured by Olympus IX73 fluorescence microscopy.

As for intracellular Fe^3+^ detection, Orn-CDs (0.5 mg ml^−1^) were added to A549 cells for 4 h as described above, followed by incubation with 200 µmol l^−1^ Fe^3+^ for further 2 h at 37°C. Before imaging, the cells were rinsed three times by phosphate-buffered saline. Images of the cells were immediately captured by an Olympus IX73 fluorescence microscope at room temperature.

## 3. Results and discussion

### 3.1. Characterization of the Orn-CDs

**Figure 1.**
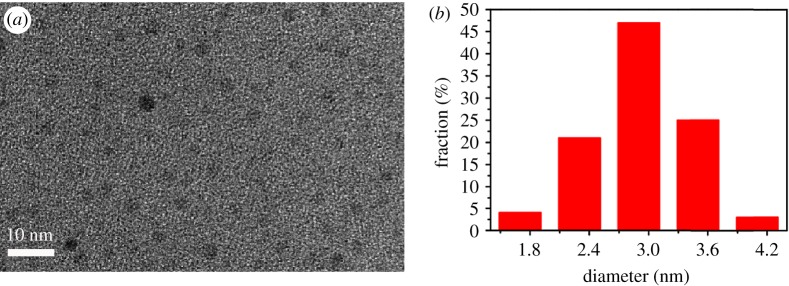
(*a*) TEM image and (*b*) particle size distribution histogram of Orn-CDs.

The shape and structure of the Orn-CDs were characterized by TEM and are displayed in figure 1. Based on the statistical analysis of 100 particles from the TEM results (figure 1*b*), the obtained Orn-CDs showed a mean diameter of 2.9 ± 0.6 nm in the range from 1.3 to 4.0 nm. It should be noted that the diffraction contrasts of Orn-CDs were very low and the lattice fringes were not found in high-resolution TEM. The corresponding selected-area electron diffraction pattern (electronic supplementary material, figure S1b) further indicated that Orn-CDs are amorphous structures [27,28].

### 3.2. Spectral properties of the Orn-CDs

**Figure 2.**
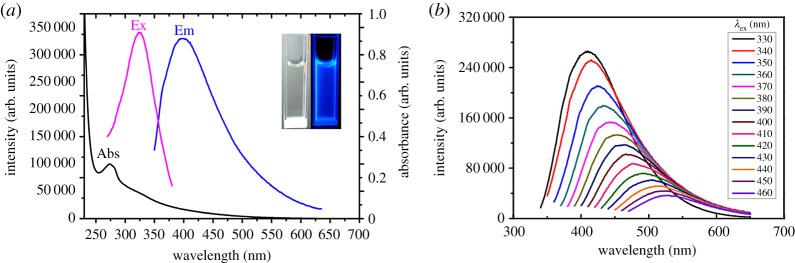
(*a*) UV–visible absorption and fluorescence spectra of Orn-CDs solution. Inset: images of the solutions of CDs. Left under sunlight; right: UV lamp illumination at 365 nm. (*b*) Emission spectra of Orn-CDs at different excitation wavelengths.

The spectral properties of Orn-CDs were investigated using UV–visible absorption and photoluminescence spectroscopy. As displayed in figure 2*a*, the UV–visible spectrum showed a strong absorption maximum at 274 nm, which corresponded to the π–π* transitions of C=C. Furthermore, Orn-CDs in aqueous solution exhibited a wide absorption peak at about 282 nm, ascribed to the n–π* transition of C=O [29]. Fluorescence spectra revealed that the optimal emission wavelengths of Orn-CDs appeared at 400 nm under excitation at 323 nm. The inset of figure 2*a* displays the digital images of Orn-CD dispersions in pure water under irradiation of visible light and UV lamp (365 nm). Similar to other related reports, the emission spectra of Orn-CDs were dependent on the excitation wavelength [28,30]. The maximum emission wavelength changed from 410 nm to 527 nm when the excitation wavelength varied from 330 nm to 460 nm (figure 2*b*). Standard quinine sulfate solutions (QY = 54%, *λ*ex = 360 nm) were used as the reference [31]. The fluorescence quantum yield of Orn-CDs was 4.77%.

FTIR and XPS experiments were carried out to investigate the structure and functional groups of Orn-CDs. As shown in electronic supplementary material, figure S2, the absorption band at 3423 cm^−1^ belonged to the stretching vibration of O–H [30], and the band at 3213 cm^−1^ was ascribed to N–H [32]. The peak at 1699 cm^−1^ was relevant to C=O bond [33,34]. The existence of carboxylic acid could be confirmed by the combination of C=O and O–H stretching vibrations. Broad band centred at 2877 cm^–1^ indicated the formation of amino group (–NH_2_ and NH_3_^+^) on the exterior of Orn-CDs [15,35].

The XPS results displayed four peaks, 531.2, 400.4, 285.0 and 197.9 eV, which were ascribed to O _1s_, N _1s_, C _1s_ and Cl _2p_, respectively (figure S3a). The high-resolution spectra of C _1s_, N _1s_, and O _1s_ are demonstrated in electronic supplementary material, figure S3b–d. The C _1s_ spectrum could be deconvoluted into three components. Peaks at 284.8, 285.9 and 287.9 eV could be ascribed to C=C, C–O and C=O groups, respectively (electronic supplementary material, figure S3b) [30,36]. The N _1s_ spectrum displayed two peaks, 399.6 eV and 401.2 eV, indicating the existence of C–N–C and N–H bonds, respectively (electronic supplementary material, figure S3c) [37]. The O _1s_ spectrum showed two main peaks, 531.1 eV and 532.2 eV, owing to C–O and C=O groups, respectively (electronic supplementary material, figure S3d) [38,39]. Combining the FTIR and XPS results, we validated the existence of hydroxyl, amino and carboxyl groups on the exterior of Orn-CDs.

### 3.3. Stability of the Orn-CDs

**Figure 3.**
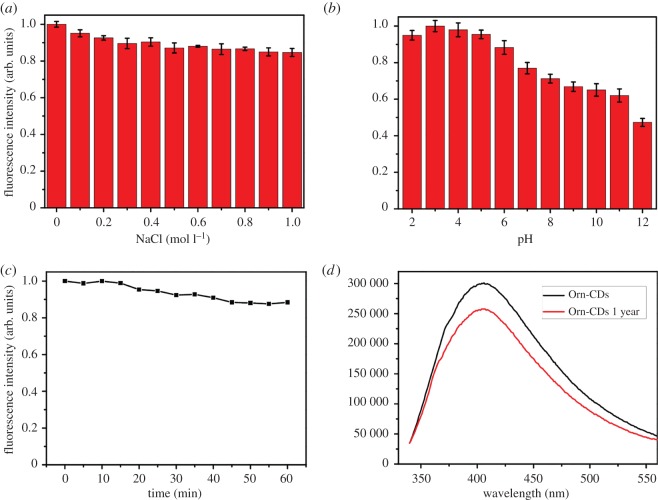
(*a*) Influence of the concentration of NaCl on the emission intensity of Orn-CDs. (*b*) Influence of the solution pH on the emission intensity of Orn-CDs. (*c*) Effect of exposure time under UV light (365 nm, 8 W) on the emission intensity of Orn-CDs. (*d*) Fluorescence spectra of fresh Orn-CDs and Orn-CDs (1 mg ml^−1^ in aqueous solutions) after 1 year of storage in air at room temperature.

The effects of parameters such as different NaCl concentration, various pH, prolonged exposure to UV light, and storage in air at room temperature on the stability of Orn-CDs were examined. As illustrated in figure 3*a,c*, the fluorescence intensity was almost unchanged at different ionic strengths in NaCl solution (up to 1.0 mol l^−1^) or under consecutive illumination for 60 min. Orn-CDs displayed pH-dependent fluorescence behaviour and relatively stable emission intensity within the pH range from 2.0 to 9.0 (figure 3*b*), which may be attributed to the functional groups, including hydroxyl, amino and carboxyl groups of Orn-CDs [34,35]. The fluorescence intensity of Orn-CDs had no obvious decrease (figure 3*d*) after being stored 1 year at ambient temperature, similar to other CDs derived from amino acids [27]. All these merits would make Orn-CDs a promising candidate for biological applications.

### 3.4. Determination of Fe^3+^ and ascorbic acid using Orn-CDs

To acquire the best result for Fe^3+^ and ascorbic acid detection, the influences of (A) solution pH; (B) concentration of Orn-CDs; and (C) incubation time were surveyed and optimized. Electronic supplementary material, figure S4a, depicts the influence of solution pH on the fluorescence quenching capacity of Orn-CDs by Fe^3+^ and fluorescence recovering efficiency by ascorbic acid in Tris–HCl buffer solution. The following equations describe how the two efficiencies were evaluated:

$$\mathrm{Ef}f_{q}\;(\text{\%})=\frac{F_{0}-F}{F_{0}},$$

$$\mathrm{Ef}f_{r}\;(\text{\%})=\frac{F_{r}-F}{F_{0}-F},$$

where *F*_0_ and *F* are the emission intensities of Orn-CDs at 404 nm before and after the addition of Fe^3+^, respectively. *F*_*r*_ is the recovered fluorescence intensity of Orn-CDs at 404 nm after the addition of ascorbic acid. As displayed, both the Eff*_q_* and Eff*_r_* increased gradually along with the increment of pH from 3.0 to 5.5, then decreased from 5.5 to 8.0. Thus, pH = 5.5 was set as the suitable pH for further experiments. As presented in electronic supplementary material, figure S4b, the Eff_*q*_ had hardly changed when the concentration of Orn-CDs varied from 0.05 mg ml^−1^ to 0.25 mg ml^−1^; the Eff_*r*_ increased slightly with the increment of Orn-CDs from 0.05 mg ml^−1^ to 0.15 mg ml^−1^, then decreased slowly from 0.15 mg ml^−1^ to 0.25 mg ml^−1^. Thus, we chose 0.15 mg ml^−1^ as the probe concentration. Electronic supplementary material, figure S4c, shows that the emission intensity of Orn-CDs reduced gradually in the presence of Fe^3+^ (20 µmol l^−1^), and then became relatively stable after 20 min. Hence, 20 min was set as the reaction time for Fe^3+^ detection. As displayed in electronic supplementary material, figure S4d, in the presence of ascorbic acid, the emission intensity of the mixture of Orn-CDs/Fe^3+^ increased slowly and reached the apex after 25 min. Thus, 25 min was adopted as the reaction time for ascorbic acid determination.

**Figure 4.**
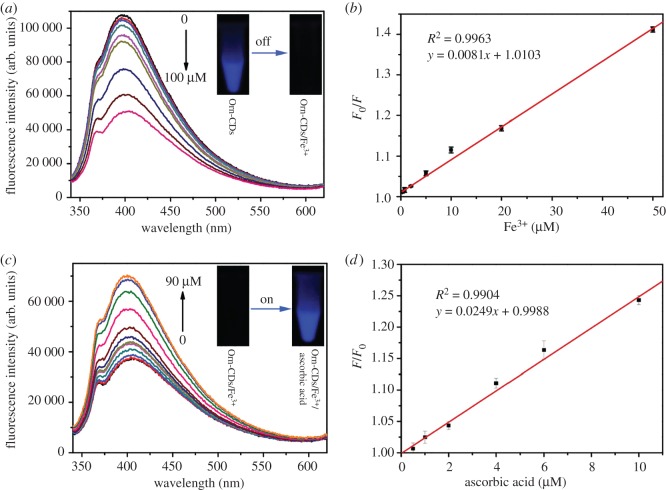
(*a*) Fluorescence response of Orn-CDs (0.15 mg ml^−1^ in 20 mmol l^−1^ Tris–HCl buffer) toward various concentrations of Fe^3+^ from 0 to 100 µmol l^−1^. (*b*) *F*_0_/*F* value versus the concentration of Fe^3+^ (from left to right: 0.3, 2, 5, 10, 20, 50 µmol l^−1^). *F* and *F*_0_ are the emission intensities of Orn-CDs (0.15 mg ml^−1^ in Tris–HCl solution) at 404 nm before and after the addition of Fe^3+^, respectively. (*c*) Fluorescence emission spectra of Orn-CDs/Fe^3+^ sensing system (0.15 mg ml^−1^ Orn-CDs and 200 µmol l^−1^ Fe^3+^ in 20 Tris–HCl solution) after the addition of various concentrations of ascorbic acid from 0 to 90 µmol l^−1^. (*d*) Relationship between *F*/*F*_0_ and the amount of ascorbic acid (from left to right: 0.5, 1, 2, 4, 8, 10 µmol l^−1^). *F* and *F*_0_ denote the emission intensities of Orn-CDs/Fe^3+^ system at 404 nm in the presence and absence of ascorbic acid, respectively.

We further examined the sensing ability of Orn-CDs to Fe^3+^ under optimum conditions. The emission intensity at 404 nm reduced slowly with the increment of Fe^3+^ (figure 4*a*). The value of *F*_0_/*F* showed a good linear correlation with the Fe^3+^ concentrations from 0.3 to 50 µmol l^−1^ (figure 4*b*, *R*^2^ = 0.9963). Here, *F*_0_ and *F* denote the fluorescence intensities of Orn-CDs at 404 nm before and after the addition of Fe^3+^ ions, respectively. The limit of detection was 95.6 nmol l^−1^ (3*σ*/*k*, wherein *σ* represents the standard deviation of blank solution and *k* denotes the slope of the linear calibration plot), which was similar to the previously reported results by using CDs as the sensor (electronic supplementary material, table S1).

The selectivity of this probe was investigated by screening Fe^3+^ (50 µmol l^−1^) as well as other different metal ions at a concentration of 2 mmol l^−1^ such as Na^+^, K^+^, Ca^2+^, Cu^2+^, Mg^2+^, Fe^2+^, Zn^2+^, Ba^2+^, Pb^2+^, Al^3+^, Ni^2+^, Co^2+^, Cr^3+^, Cd^2+^, Hg^2+^ and Mn^2+^. As illustrated in electronic supplementary material, figure S5, Orn-CDs showed a highest selectivity for Fe^3+^ detection among the other different metal ions, indicating its potential application for Fe^3+^ detection.

Ascorbic acid could convert Fe^3+^ to Fe^2+^ via the oxidation/reduction reaction. As expected, the quenched fluorescence of Orn-CDs/Fe^3+^ system could be restored by ascorbic acid (electronic supplementary material, figure S6). Furthermore, ascorbic acid showed negligible influence on the emission intensity of Orn-CDs (electronic supplementary material, figure S7). Figure 4*c* shows that the emission intensities of Orn-CDs/Fe^3+^ were progressively enhanced in the presence of ascorbic acid. The value of *F/F*_0_ displayed a good linear correlation with the ascorbic acid concentrations from 0.5 to 10 µmol l^−1^ (figure 4*d*, *R*^2^ = 0.9904). Here, *F* and *F*_0_ denote the emission intensities of Orn-CDs at 404 nm before and after the addition of ascorbic acid, respectively. The limit of detection was 137 nmol l^−1^ (calculated by the formula of 3*σ*/*k*), similar to the previously reported results by using CDs as the sensor (electronic supplementary material, table S2).

To evaluate the selectivity of Orn-CDs/Fe^3+^ sensing system for the detection of ascorbic acid (10 µmol l^−1^), the potential interference of metal cations (Ca^2+^, Mg^2+^, K^+^, Na^+^), amino acids (l-cysteine, glycine, glutamic acid, serine, threonine, histidine), glutathione and bovine serum albumin was investigated. The concentration of each interferent was 100 µmol l^−1^. Electronic supplementary material, figure S8, shows that the emission intensity of Orn-CDs/Fe^3+^ increased remarkably upon the addition of ascorbic acid. The most interfering analytes showed no obvious influence on the emission intensity of Orn-CDs/Fe^3+^. This result demonstrated the excellent selectivity of Orn-CDs/Fe^3+^ for ascorbic acid detection.

### 3.5. Potential mechanism of Orn-CDs for the detection of Fe^3+^

The potential mechanism of fluorescence quenching of Orn-CDs by Fe^3+^ was also investigated. As depicted in electronic supplementary material, figure S9, the absorption band in the UV–visible absorption spectra did not change in the presence of Fe^3+^, indicating that the quenching process by Fe^3+^ was not static quenching [40]. To further reveal the mechanism of Orn-CDs for the detection of Fe^3+^, the Stern–Volmer equation of Orn-CDs in the presence of Fe^3+^ was analysed as *F*_0_/*F* = 1 + *K*_sv_ [Q], where *F*_0_ and *F* are the emission intensities of Orn-CDs at 404 nm before and after the addition of Fe^3+^, respectively. *K*_sv_ is the quenching constant of the equation and [Q] is the concentration of Fe^3+^. *K*_sv_ is calculated as 8.1 × 10^3^ mol^−1^ l. Electronic supplementary material, figure S10 and table S3, shows that the fluorescence lifetime of Orn-CDs is reduced from 9.21 ns to 7.30 ns in the presence of Fe^3+^. The reduction of fluorescence lifetime suggested the occurrence of dynamic quenching [30,41]. The quenching process was further considered to be dynamic because *F*_0_/*F* = *τ*_0_/*τ*, where *τ*_0_ and *τ* are the fluorescence lifetimes of Orn-CDs at 404 nm before and after the addition of Fe^3+^, respectively [37,42]. The absorption peak of Orn-CDs/Fe^3+^ at 274 nm reduced progressively upon the addition of ascorbic acid (electronic supplementary material, figure S9), indicating the combination of Fe^3+^ and Orn-CDs was suppressed and the recovery of Orn-CDs emission by ascorbic acid.

### 3.6. Determination of Fe^3+^ and ascorbic acid in human serum and urine specimens

The feasibility of Orn-CDs as a sensor for detection of Fe^3+^ in human serum and urine samples was also investigated. As depicted in electronic supplementary material, table S4, the relative standard deviation was less than 3.80% (*n* = 3) with satisfactory recoveries (94.1–108.4%) for Fe^3+^ detection.

The practical application of the mixture of Orn-CDs and Fe^3+^ for ascorbic acid detection was performed in human urine specimens. As shown in electronic supplementary material, table S5, the relative standard deviation was less than 3.47% (*n* = 3) with satisfactory recoveries (94.0–106.8%) for ascorbic acid detection. The results validated the reliability and practicability of the Orn-CDs/Fe^3+^-based probe for ascorbic acid detection in real specimens.

### 3.7. Intracellular imaging of Fe^3+^

**Figure 5.**
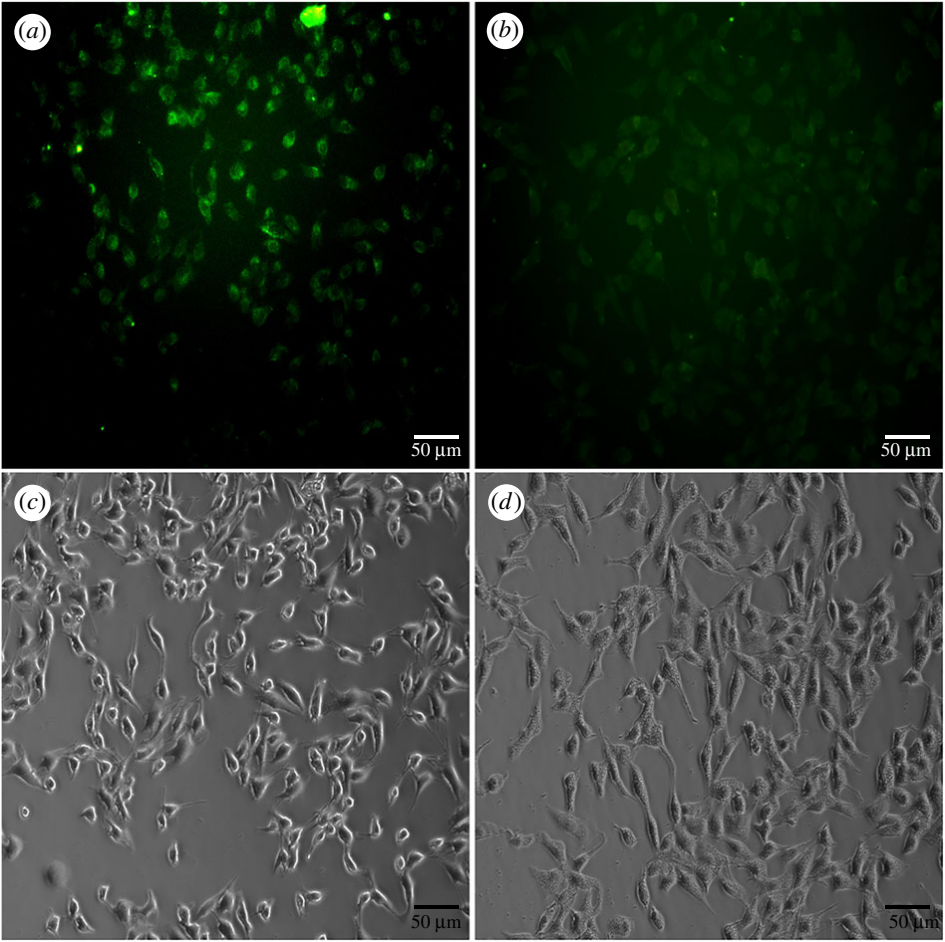
(*a*) Fluorescence microscopy images of A549 cells after incubation with 0.5 mg ml^−1^ Orn-CDs for 4 h at 37°C, (*b*) further incubated with 200 µmol l^−1^ Fe^3+^ for another 2 h at 37°C. (*a,b*) Fluorescence images and (*c,d*) their corresponding bright-field microscope images, respectively. The scale bar is 50 µm.

The biocompatibility of Orn-CDs against A549 cells was investigated by MTT assay. Electronic supplementary material, figure S11, shows that the cell viability was more than 82% after the addition of 1 mg ml^−1^ of Orn-CDs for 24 h. The results indicated that Orn-CDs may be suitable for Fe^3+^ sensing in living cells. As displayed in figure 5*a*, the cytoplasma of A549 cells emitted green fluorescence after treatment with Orn-CDs for 4 h. The intracellular fluorescence of A549 cells became weaker after the addition of 200 µmol l^−1^ Fe^3+^ for another 2 h at 37°C (figure 5*b*), indicating its potential application for intracellular Fe^3+^ sensing.

Compared to natural amino acids, ornithine contains an additional amine group which would endow the resulting CDs with extra positive charge. Our preliminary results showed that zeta potential of Orn-CDs aqueous solution was positive and exhibited antibacterial capacity (data not shown), similar to related reports by using polyamine compound (spermidine) as the precursor to fabricate CDs [43]. More in-depth research is under way.

## 4. Conclusion

Orn-CDs were prepared via a one-step hydrothermal strategy using l-ornithine hydrochloride as the sole precursor. The prepared Orn-CDs showed excellent fluorescence properties, high yield and low cytotoxicity. Owing to the selective recognition and sensitive fluorescent response of Fe^3+^ by Orn-CDs, they were successfully used for Fe^3+^ determination in biological samples (human serum and urine). Moreover, ascorbic acid could recover the fluorescence of Orn-CDs quenched by Fe^3+^. Accordingly, the platform was used for the quantification of ascorbic acid in real urine specimens. Orn-CD-based sensing platform showed its potential practical applications in clinical diagnosis and biological fields because it is cost-effective, easily scalable and can be used without additional functionalization and sample pre-treatment.

## Supplementary Material

Electronic Supplementary Material

## Data accessibility

Data are available from the Dryad Digital Repository: http://dx.doi.org/10.5061/dryad.6c9143t [44].
