# Supplementary material for: Nitrogen and chlorine co-doped carbon dots as probe for sensing and imaging in biological samples
Source: R Soc Open Sci. 2019 Jan 23;6(1):181557. doi: 10.1098/rsos.181557 (PMC6366224; doi:10.1098/rsos.181557)
Supplement: Electronic Supplementary Material [file rsos181557supp1.docx]

**Electronic Supplementary Material**

**Nitrogen and** **chlorine co-doped carbon dots as probe for sensing and imaging in biological samples**

Jin Li^a,1^, Kai Tang^b,1^, Jianxin Yu^b^, Hanqin Wang^b^, Mingli Tu^b^, Xiaobo Wang^a,b*^

^a^ Department of reproductive medicine, Suizhou Hospital, Hubei University of

Medicine, 60 Longmen Street, Suizhou 441300, China

^b^ Center for translational medicine, Suizhou Hospital, Hubei University of Medicine,

8 east central park road, Suizhou 441300, China

^1^ These authors contributed equally to this work

***Corresponding Author**

Xiaobo Wang

<Tel:86-0722-3252551>

Fax:86-0722-3252400

E-mail: [wangxiaobo78@126.com](mailto:wangxiaobo78@126.com)

**Figures, Tables.**

**Fig. S1** TEM images (a) of Orn-CDs (inset: the HRTEM image of the Orn-CDs) ,

and the corresponding SAED image (b) and XRD patterns of Orn-CDs (c).

**Fig. S2** FTIR spectra of the prepared Orn-CDs.

**Fig. S3** XPS survey spectra (a), high resolution XPS spectra of C _1s_ (b), N _1s_ (c), O _1s_

(d) of the prepared Orn-CDs.

**Fig. S4** The influence of pH value, concentrations of Orn-CDs, incubation time of

Orn-CDs based sensing system on the detection of Fe^3+^ and ascorbic acid.

**Table S1** Comparison of Orn-CDs and other carbon dots based probes for Fe^3+^ detection.

**Fig. S5** Selectivity of Orn-CDs toward different metal ions.

**Fig. S6** Photographs of Orn-CDs upon addition of Fe^3+^and ascorbic acid.

**Fig. S7** Fluorescence spectrum of Orn-CDs before and after addition of ascorbic acid.

**Table S2** Comparison of Orn-CDs and other probes for ascorbic acid detection.

**Fig.S8** Selectivity of Orn-CDs/Fe^3+^ system toward ascorbic acid.

**Fig. S9** UV-vis spectrum of Orn-CDs in the presence of Fe^3+^and ascorbic acid.

**Fig. S10** Fluorsecence decays of the prepared Orn-CDs upon addition of Fe^3+^.

**Table S3** Double-exponential fitting of Orn-CDs and Orn-CDs /Fe^3+^ decay curves.

**Table S4** Fe^3+^ determination results in human serum and urine samples.

**Table S5** Ascorbic acid determination results in human urine samples.

**Fig. S11** Viability of the prepared Orn-CDs against A549 cells.

**
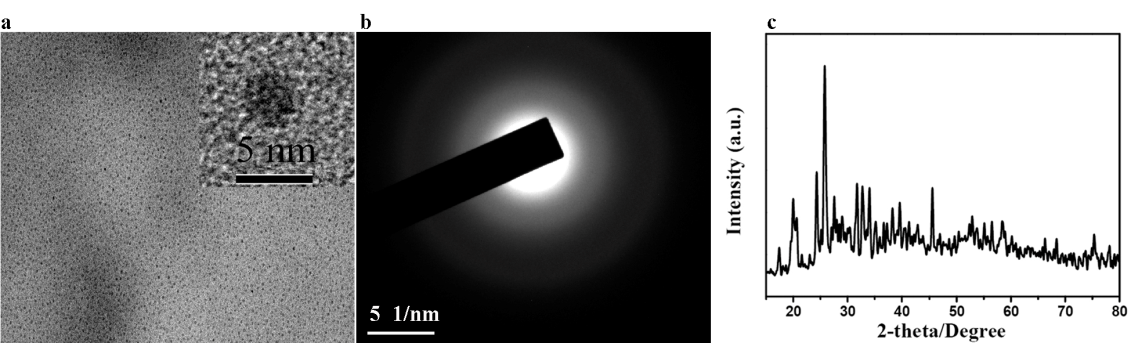
**

**Fig. S1** TEM images (a) of Orn-CDs (inset: the HRTEM image of the Orn-CDs),

and the corresponding SAED image (b) and XRD patterns of Orn-CDs (c).


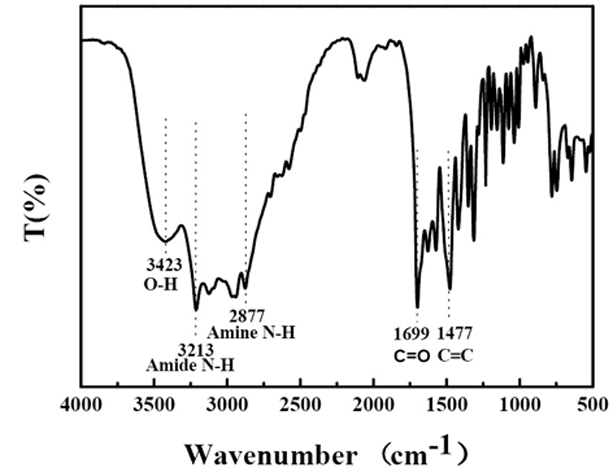


**Fig. S2** FTIR spectra of the prepared Orn-CDs.


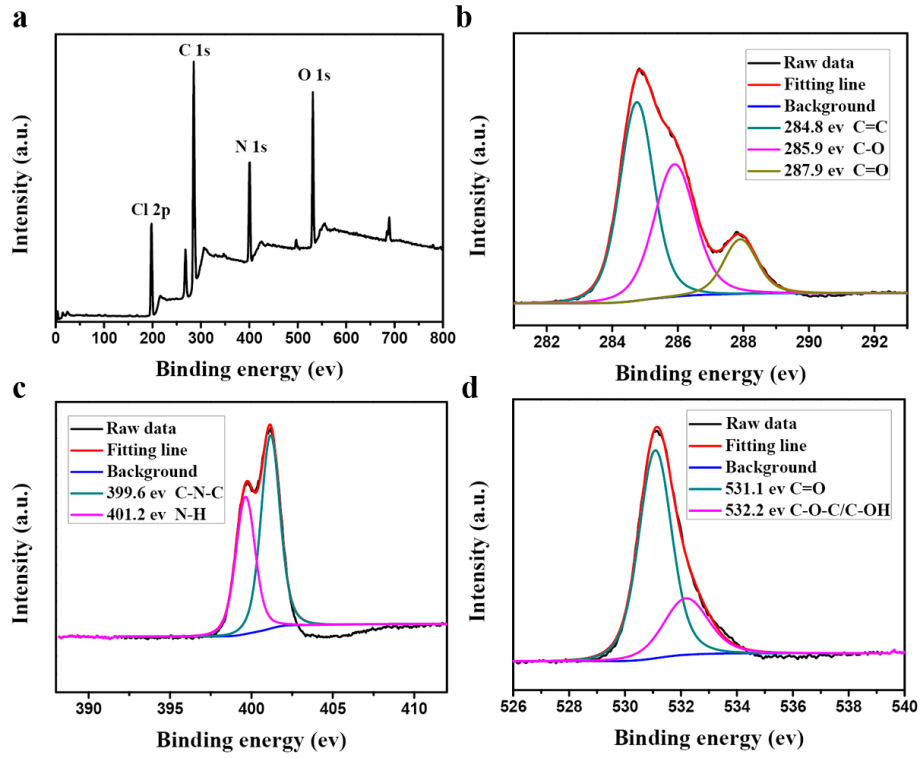


**Fig. S3** (a) XPS survey spectra, high resolution XPS spectra of C _1s_ (b), N _1s_ (c), O _1s_ (d) of the prepared Orn-CDs

**
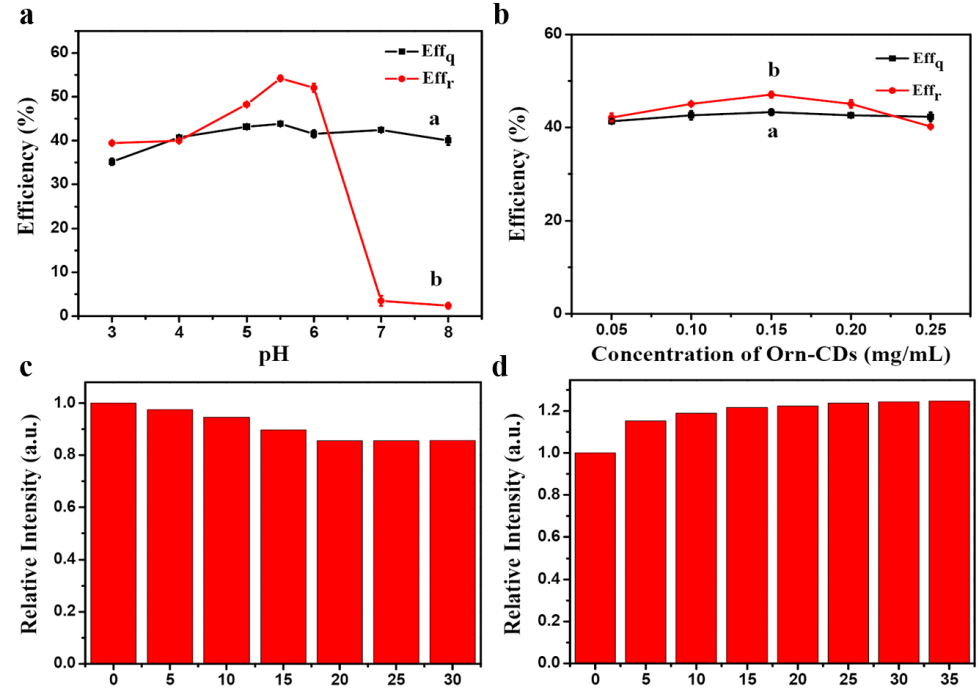
**

**Fig. S4** (a) The influence of pH value (3.0-8.0) of Tris-HCl solution (20 mmol L^-1^) on the Orn-CDs (0.15 mg mL^-1^) fluorescence quenching efficiency (Eff_q_, black line) by 200 µmol L^-1^ Fe^3+^ and Orn-CDs (0.15 mg mL^-1^)/Fe^3+^ (200 µmol L^-1^) platform fluorescence recovering efficiency (Eff_r_, red line) after addition of 50 µmol L^-1^ ascorbic acid. (b) The effect of concentrations of Orn-CDs (from left to right : 0.05, 0.10, 0.15, 0.20, 0.25 mg mL^-1^) on the fluorescence quenching efficiency (Eff_q_, black line) by 200 µmol L^-1^ Fe^3+^ and Orn-CDs (0.15 mg mL^-1^)/Fe^3+^ (200 µmol L^-1^) platform fluorescence recovering efficiency (Eff_r_, red line) after addition of 50 µmol L^-1^ ascorbic acid. (c) Time dependent FL response of 0.15 mg/ml Orn-CDs to 20 µmol L^-1^ Fe^3+^ in Tris-HCl solution at room temperature. (d) Time dependent fluorescence response of 0.15 mg mL^-1^ of Orn-CDs /200 µmol L^-1^ of Fe^3+^ to 10 µmol L^-1^ ascorbic acid in Tris-HCl solution at room temperature.

Table S1 Comparison of Orn-CDs and other carbon dots based probes for Fe^3+^ detection

| samples | linear range  (µmol L^-1^) | detection limit  (µmol L^-1^) | ref |
| --- | --- | --- | --- |
| water samples  human serum, urine | —  0.3-546 | 0.01  0.09 | [^1^](#_ENREF_1)  [^2^](#_ENREF_2) |
| water samples | 0.11-4.46 | 0.05 | [^3^](#_ENREF_3) |
| water samples  water samples  drinking and tap water  tap water,fruits | —  —  —  25-200 | 10  10  0.29  0.05882 | [^4^](#_ENREF_4)  [^5^](#_ENREF_5)  [^6^](#_ENREF_6)  [^7^](#_ENREF_7) |
| water, urine | 0.05-10.0 | 0.0137 | [^8^](#_ENREF_8) |
| human serum, urine | 0.3-50.0 | 0.0956 | this work |


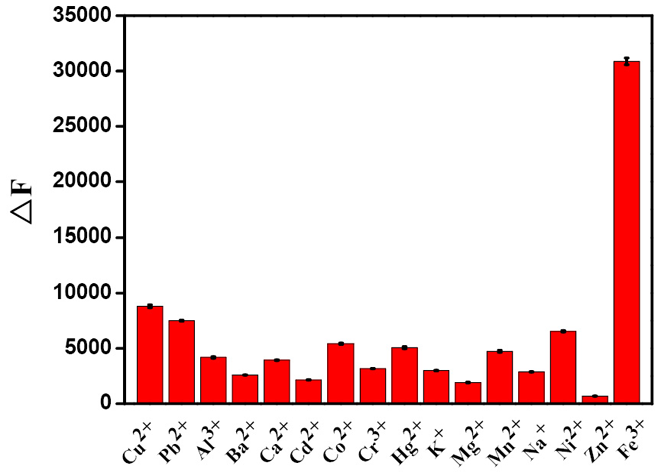


**Fig. S5** Selectivity of Orn-CDs toward different metal ions. The concentration of Orn-CDs is 0.15 mg mL^-1^ in Tris-HCl solution, 50 µmol L^-1^ for Fe^3+^ and 2 mmol L^-1^ for other metal ions.


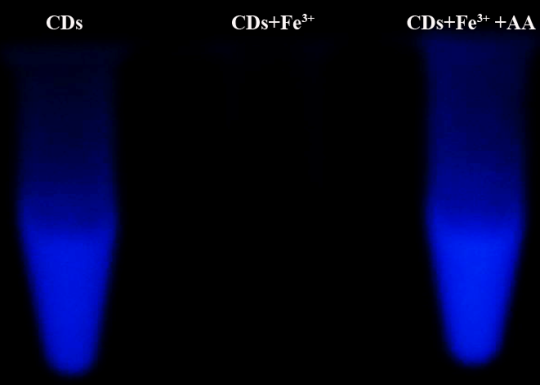


**Fig. S6** Photographs of Orn-CDs (5 mg mL^-1^) (left), Orn-CDs(5 mg mL^-1^) + Fe^3+^(10 mmol L^-1^ (middle), and Orn-CDs(5 mg mL^-1^) + Fe^3+^ (10 mmol L^-1^) + ascorbic acid (10 mmol L^-1^) (right).

**Fig. S7** Fluorescence spectra of 0.15 mg mL^-1^ of Orn-CDs before and after additon of ascorbic acid (10 µmol L^-1^).

Table S2 Comparison of Orn-CDs and other probes for ascorbic acid detection

| samples | linear range  (µmol L^-1^) | detection limit  (µmol L^-1^) | ref |
| --- | --- | --- | --- |
| fruits  human urine  juice  human serum  human blood plasma  water  human urine | 25-300  0.2-11.0  1-90  0.15-15.0  1.5-10  8-100  0.5-10.0 | 0.236  0.01  0.018  0.105  0.2  2.4  0.137 | [^7^](#_ENREF_7)  [^8^](#_ENREF_8)  [^9^](#_ENREF_9)  [^10^](#_ENREF_10)  [^11^](#_ENREF_11)  [^12^](#_ENREF_12)  this work |


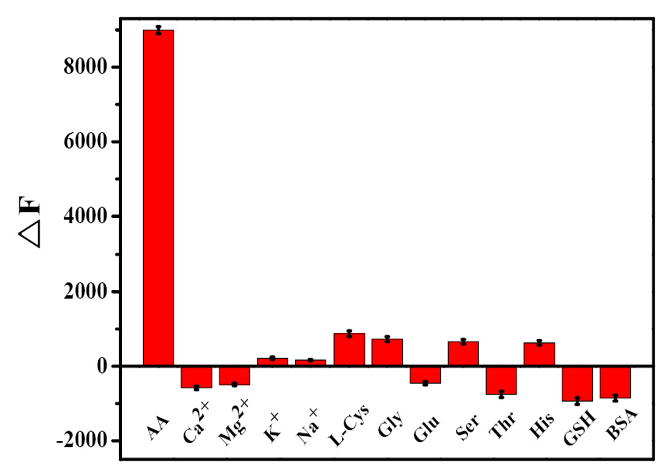


**Fig. S8** Selectivity of Orn-CDs/Fe^3+^ system toward ascorbic acid. The concentration of Orn-CDs is 0.15 mg mL^-1^ in Tris-HCl solution, 200 µmol L^-1^ of Fe^3+^and 10 µmol L^-1^ of ascorbic acid .The concentration of other interference analytes is 100 µmol L^-1^.


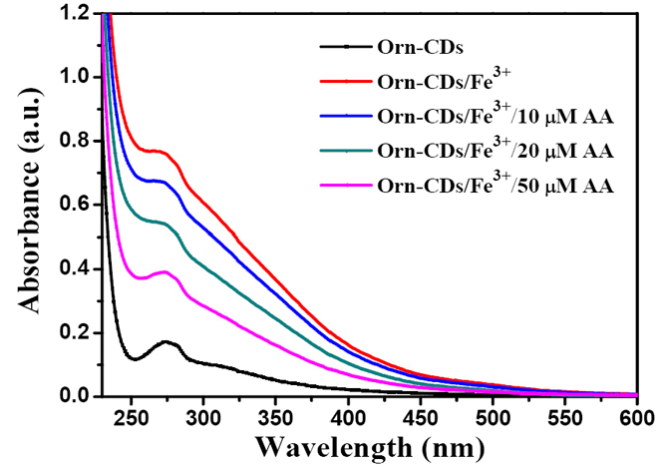


**Fig. S9** UV-vis spectra of the prepared Orn-CDs (0.15 mg mL^-1^, black line), 0.15 mg mL^-1^ Orn-CDs/200 µmol L^-1^ of Fe^3+^ (red line), and 0.15 mg mL^-1^ Orn-CDs/200 µmol L^-1^ Fe^3+^/AA (blue line for 10 µmol L^-1^ AA, green line for 20 µmol L^-1^ AA, purple line for 50 µmol L^-1^ AA respectively)

Fluorescence decay curves were performed in a time–correlated-single-photo- counting (TCSPC) system from FL980 spectrometer under excitation at 326 nm. Data were fit by using the bi-exponential function in equation (1).

  $Y(t)=\alpha_{1}\exp(-t/\tau_{_{1}})+\alpha_{2}\exp(-t/\tau_{2})$ (1)

α_1_ and α_2_ were the fractional contributions of time-resolved decay lifetime of $\tau_{1}$and$\tau_{2}$.


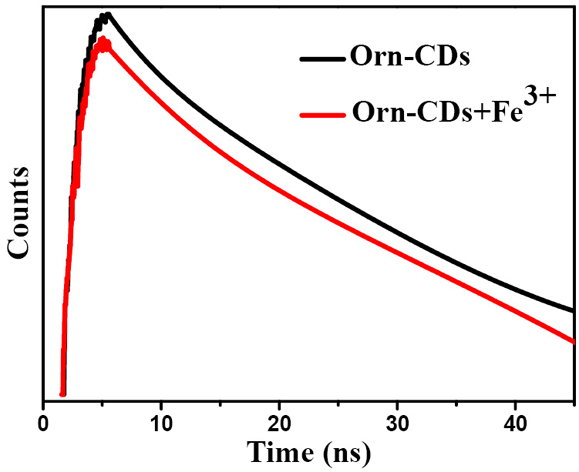


**Fig. S10** Fluorescence decays of the prepared Orn-CDs (0.15 mg mL^-1^, black curve) upon addition of 100 µmol L^-1^ Fe^3+^ (red curve) at excitation/emission wavelengths (λ_ex_/λ_em_) of 326/404 nm.

**Table S3.** Double-exponential fitting of Orn-CDs and Orn-CDs /Fe^3+^ decay curves.

| Sample name | Orn-CDs | Orn-CDs /Fe^3+^ |
| --- | --- | --- |
| *τ_1_*(ns)/*A_1_* (%) | 3.5841/36.3 | 2.4074/21.24 |
| *τ_2_*(ns)/*A_2_* (%) | 12.4129/63.7 | 8.6186/78.76 |
| Average *τ* (ns) | 9.21 | 7.30 |

**Table S4**. Fe^3+^ determination results in human serum and urine samples.

| Sample | Added  (µmol L^-1^) | Found  (µmol L^-1^) | Recovery (%, n=3) | RSD (%, n=3) |
| --- | --- | --- | --- | --- |
| Serum | 0.50 | 0.542 | 108.4 | 2.41 |
|  | 5.00 | 5.126 | 102.5 | 3.80 |
|  | 20.00  50.00 | 18.811  53.837 | 94.1  107.7 | 1.48  2.30 |
| Urine | 0.50 | 0.480 | 95.9 | 1.59 |
|  | 5.00 | 4.921 | 98.4 | 3.67 |
|  | 20.00  50.00 | 20.468  53.236 | 102.3  106.5 | 2.17  3.75 |

**Table S5**. Ascorbic acid determination results in human urine samples.

| Sample | Added  (µmol L^-1^) | Found  (µmol L^-1^) | Recovery (%, n=3) | RSD (%, n=3) |
| --- | --- | --- | --- | --- |
| Urine 1 | 0.50 | 0.470 | 94.0 | 2.21 |
|  | 2.00 | 1.887 | 94.4 | 3.10 |
|  | 5.00  10.00 | 5.166  9.878 | 103.3  98.8 | 2.12  3.16 |
| Urine 2 | 0.50 | 0.474 | 94.9 | 2.07 |
|  | 2.00 | 2.136 | 106.8 | 2.78 |
|  | 5.00  10.00 | 4.831  10.490 | 96.6  104.9 | 3.47  2.66 |

**Cell viability assay**

The biocompatibility of Orn-CDs against A549 cells were investigated by MTT assay. After seeding A549 in 96-well plate at 1×10^4^/well and cultured for 24 h at 37°C under 5% CO_2_, the supernatant was discarded and the cells were washed three times with PBS, subsequently, different concentration (0, 0.1, 0.2, 0.3, 0.4, 0.5, 0.6, 0.7, 0.8, 0.9, 1.0 mg mL^-1^ ) of the Orn-CDs in DMEM medium were added (100 μL/well) and clutured for furthe 24 h at 37°C. Finally, MTT (5 mg mL^-1^, 20 μL/well) was added and incubated for another 4 h. After removing the culture medium, DMSO (150 μL/well) was added and shaken at 37°C for 10 min. A microplate reader (Bio Tek Epoch) was used to measure the absorbance of each sample at 490 nm. The data represent the mean ± standard deviation of four independent experiments. The cell viability was expressed as the below formula:

$cell viability\left( \% \right)= \frac{\mathrm{OD}_{\mathrm{treated}}}{\mathrm{OD}_{\mathrm{control}}} \times100\%$

where OD_control_ was acquired before addition of Orn-CDs and OD_treated_ was acquired in the presence of Orn-CDs.


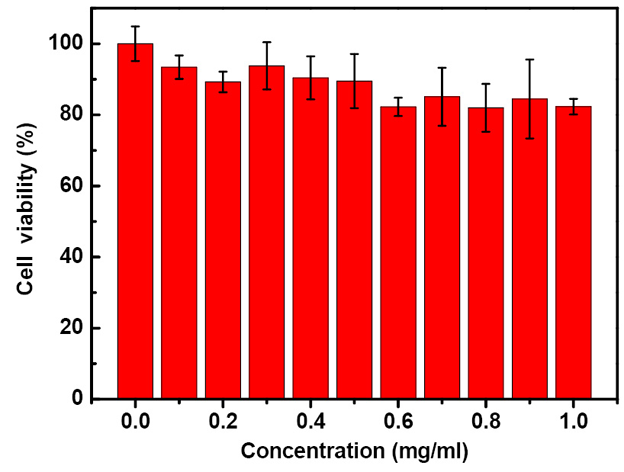


**Fig. S11** Viability of the prepared Orn-CDs against A549 cells.

References

1. H. Huang, C. Li, S. Zhu, H. Wang, C. Chen, Z. Wang, T. Bai, Z. Shi and S. Feng, *Langmuir*, 2014, **30**, 13542-13548. [(doi:10.1021/la503969z)](https://doi.org/10.1021/la503969z)

2. Y. Liu, W. Duan, W. Song, J. Liu, C. Ren, J. Wu, D. Liu and H. Chen, *ACS applied materials & interfaces*, 2017, **9**, 12663-12672. [(doi:10.1021/acsami.6b15746)](https://doi.org/10.1021/acsami.6b15746)

3. P. Karfa, E. Roy, S. Patra, S. Kumar, A. Tarafdar, R. Madhuri and P. K. Sharma, *RSC Advances*, 2015, **5**, 58141-58153. [(doi:10.1039/c5ra09525e)](https://doi.org/10.1039/c5ra09525e)

4. G. He, M. Xu, M. Shu, X. Li, Z. Yang, L. Zhang, Y. Su, N. Hu and Y. Zhang, *Nanotechnology*, 2016, **27**, 395706. [(doi:10.1088/0957-4484/27/39/395706)](https://doi.org/10.1088/0957-4484/27/39/395706)

5. Z. Wang, C. Xu, Y. Lu, X. Chen, H. Yuan, G. Wei, G. Ye and J. Chen, *Sensors and Actuators B: Chemical*, 2017, **241**, 1324-1330. [(doi:10.1016/j.snb.2016.09.186)](https://doi.org/10.1016/j.snb.2016.09.186)

6. H.-J. Cheng, C.-L. Kao, Y.-F. Chen, P.-C. Huang, C.-Y. Hsu and C.-H. Kuei, *Microchimica Acta*, 2017, **184**, 3179-3187. [(doi:10.1007/s00604-017-2336-7)](https://doi.org/10.1007/s00604-017-2336-7)

7. F. Du, X. Gong, W. Lu, Y. Liu, Y. Gao, S. Shuang, M. Xian and C. Dong, *Talanta*, 2018, **179**, 554-562. [(doi:10.1016/j.talanta.2017.11.030)](https://doi.org/10.1016/j.talanta.2017.11.030)

8. M. Shamsipur, K. Molaei, F. Molaabasi, M. Alipour, N. Alizadeh, S. Hosseinkhani and M. Hosseini, *Talanta*, 2018, **183**, 122-130. [(doi:10.1016/j.talanta.2018.02.042)](https://doi.org/10.1016/j.talanta.2018.02.042)

9. X. Kong, Y. Gong and Z. Fan, *Journal of fluorescence*, 2016, **26**, 1755-1762. [(doi:10.1007/s10895-016-1867-3)](https://doi.org/10.1007/s10895-016-1867-3)

10. W.-J. Niu, D. Shan, R.-H. Zhu, S.-Y. Deng, S. Cosnier and X.-J. Zhang, *Carbon*, 2016, **96**, 1034-1042. (doi:10.1016/j.carbon.2015.10.051)

11. X. Wang, P. Wu, X. Hou and Y. Lv, *The Analyst*, 2013, **138**, 229-233. [(doi:10.1039/c2an36112d)](https://doi.org/10.1039/c2an36112d)

12. C. Mi, T. Wang, P. Zeng, S. Zhao, N. Wang and S. Xu, *Analytical Methods*, 2013, **5**, 1463-1468. [(doi:10.1039/c3ay26387h)](https://doi.org/10.1039/c3ay26387h)
